# Toll-like receptor 2 is present in the microenvironment of oral squamous cell carcinoma

**DOI:** 10.1038/sj.bjc.6606057

**Published:** 2010-12-21

**Authors:** L K Ng, A M Rich, H M Hussaini, W M Thomson, A L Fisher, L S Horne, G J Seymour

**Affiliations:** 1 Immunopathology Group, Sir John Walsh Research Institute, Faculty of Dentistry, University of Otago, PO Box 647, Dunedin 9054, New Zealand

**Keywords:** toll-like receptor 2, dysplasia, hyperplasia, oral squamous cell carcinoma

## Abstract

**Background:**

The aim of this study was to investigate the expression of toll-like receptor 2 (TLR2) on cells associated with oral squamous cell carcinoma, epithelial dysplasia and irritative hyperplasia, using immunohistochemistry.

**Results:**

More immune cells expressed TLR2 in carcinoma and dysplasia than in hyperplasia (*P*<0.001). No hyperplastic samples showed positive TLR2 staining on keratinocytes, whereas keratinocytes in 64% of cases of carcinoma and 74% of cases of dysplasia were TLR2 positive.

**Conclusion:**

Positive TLR2 expression in the microenvironment suggests activation of immune surveillance against the altered epithelium, whereas TLR2 expression by malignant keratinocytes may be indicative of resistance to apoptosis as a pro-survival mechanism.

The connective tissue adjacent to oral squamous cell carcinoma (OSCC), epithelial dysplasia (ED) and in irritative hyperplasia (IH) is infiltrated by chronic inflammatory cells (CIC) and may exhibit increased vascularity with numerous endothelial cell (EC)-lined vessels. The CIC and EC communicate with other cells in the vicinity through inflammatory mediators and receptors such as toll-like receptors (TLRs). Toll-like receptor 2 is a type I transmembrane protein, which recognises a wide variety of pathogen-associated molecular patterns (PAMPs) from exogenous pathogens, as well as endogenous damage-associated molecular patterns (DAMPs) including heat shock proteins and high-mobility group box 1 protein (HMGB1) (Sato *et al*, 2009).

Toll-like receptors share a common signalling pathway through myeloid differentiation protein 88, nuclear factor *κ*-light-chain enhancer of activated B cells (NF-*κ*B) and mitogen-associated protein kinase, eventually leading to the production of type 1 interferon and an immune regulatory response (Akira and Takeda, 2004; Kumagai *et al*, 2008). Toll-like receptor expression induces reactive oxygen species and nitrogen intermediates, activates the cytokine network, initiates signal transduction cascades, increases phagocytic efficacy, is associated with maturation of dendritic cells and activates apoptotic pathways (Akira *et al*, 2001; Liu, 2006; Wang *et al*, 2008).

Research relating to TLRs has concentrated primarily on immune cells, but they are also expressed on normal and chronically inflamed epithelial cells of skin and mucosal surfaces (Sato *et al*, 2009). Further, some epithelial tumour cells have been shown to express TLRs and this may confer advantage to the tumour cell (Szczepanski *et al*, 2007, 2009; Sato *et al*, 2009).

In this study, we investigated TLR2 expression by keratinocytes and other cells in the microenvironment of human OSCC, ED and IH.

## Materials and methods

Samples were selected from archival formalin fixed paraffin-embedded tissues accessioned in the Medlab Dental Oral Pathology Diagnostic Service, Faculty of Dentistry, University of Otago. Histological reports were reviewed and tissues from 50 cases of OSCC, 50 white lesions with histological evidence of epithelial dysplasia and 50 oral mucosal hyperplastic lesions were obtained. Human fibrous epulis and tonsillar tissues were used as negative and positive controls.

**Figure 1:**
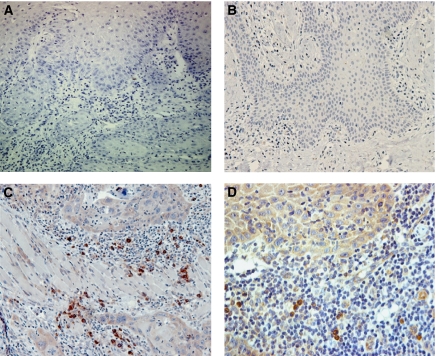
Photomicrographs of OSCC isotype control (**A**) and irritative hyperplasia (**B**) demonstrating no TLR 2 expression in the epithelium or on inflammatory cells in the connective tissue. The invading epithelial islands of an OSCC demonstrate weakly (**C**) and strongly (**D**) positive-TLR 2 expression. Some inflammatory cells in the adjacent fibrous connective tissue show strong positivity in both (**C**) and (**D**).

Immunohistochemical staining was performed using a monoclonal mouse antibody against TLR2.1 (sc-21759, Santa Cruz Biotechnology, Santa Cruz, CA, USA) and mouse avidin–biotin complex (sc-2017, Santa Cruz Biotechnology) with a dilution of 1 : 50. The sections were processed manually in the routine manner after being heated with sodium citrate 2.94 g l^−1^ at 90°C for 10 min for antigen retrieval. A sample known to be positive for TLR2 was included in each run, as was a negative control where the primary antibody was replaced by isotype-matched mouse IgG1 (Santa Cruz Biotechnology, cat: 2025, at 1 *μ*g ml^−1^ recommended dilution) (Figure 1A). All samples were counterstained with haematoxylin.

The slides were assessed blind with light microscopy (Olympus BX50; Olympus Corp., Tokyo, Japan), by one of the authors (LKN) who had been trained and calibrated by a specialist oral pathologist (AMR). CIC and EC with dark brown nuclear and cytoplasmic staining visualised at × 200 magnification were classified as being TLR2 positive. Those with pale or no staining were considered negative. Toll-like receptor 2 expression by keratinocytes was categorised into negative, weakly positive (pale brown cytoplasmic staining) or strongly positive (dark brown cytoplasmic staining). Where there was doubt about the intensity of the staining, reference was made to photographs that had been prepared as standards. A standardised methodical microscopic evaluation of the tissue was carried out beginning at one edge of the slide and assessing every second high power field, outlined by a 1 cm square 10 × 10 eyepiece graticule. Examination continued until 1000 CIC and EC in the sub-epithelial connective tissue (IH and ED) and in the connective tissue adjacent to invading epithelial islands (OSCC) were counted. The number of these cells showing positive TLR2 expression was recorded and expressed as a percentage. Toll-like receptor 2 expression by 1000 keratinocytes, in epithelium delineated in the graticule adjacent to the connective tissue, was analysed and the cells were categorised as being negative, weakly positive or strongly positive, as we have previously reported for assessment of other antibodies in oral epithelium (Kerdpon *et al*, 1997). The reproducibility of this method was confirmed by all the slides being re-analysed by the first author 4 weeks after the completion of the main assessment and by a selection of slides being counted by another specialist oral pathologist (HMH).

Inter-observer correlation was calculated using Pearson's correlation coefficient. Statistical analyses of the experimental data was performed using Mann–Whitney's *U*-test. A comparison of TLR2 expression between all three tissues and correlation with variables was made using one-way ANOVA and *t*-test. A probability value below 0.05 was considered statistically significant.

## Results

More CIC and EC in the connective tissue of OSCC (19.9%) and ED (16.4%) expressed TLR2 by comparison with those associated with IH (7.5%) (Table 1 and Figure 1). The data were found not to be normally distributed, hence, the mean percentage for each type of tissue was calculated as mean rank (Table 1). Comparisons of mean TLR2 expression in the microenvironment were calculated using the Mann–Whitney's *U*-test in order to confirm any statistical differences. *t*-tests and one-way ANOVA confirmed that TLR2 expression in the microenvironment was not affected by patient age, patient gender, site, histological differentiation or by TLR2 expression on lesional keratinocytes. The keratinocytes in IH did not express TLR2 (Figure 1). In contrast, keratinocytes in 64% of OSCC showed weak or strong TLR2 expression, as did 74% of ED (Table 2, Figure 1).

The inter-observer correlation coefficient was 0.96 (*P*<0.05), showing significant agreement between the two observers.

## Discussion

This study clearly demonstrated higher TLR2 expression on cells in the microenvironment of OSCC and dysplasia as compared with hyperplasia. Toll-like receptor expression on inflammatory cells adjacent to tumour cells has been previously described and has been considered to be beneficial to the host by enhancing maturation of antigen-presenting cells and inhibiting tumour growth (Apetoh *et al*, 2007; Szczepanski *et al*, 2009). We suggest that another possible mechanism that may contribute to tumour suppression is TLR-induced apoptosis of the altered epithelial cells. The keratinocytes in OSCC and ED have undergone genetic mutations leading to alterations to protein products and/or alterations to chromosome structure (Califano *et al*, 1996; Tsui *et al*, 2008; Sheu *et al*, 2009) and could be perceived as ‘foreign’ by the immune system. This might result in high TLR2 expression by the CIC and EC in the vicinity, as found in this study, and lead to TLR-induced apoptosis in these altered tissues (Huang *et al*, 2005, 2007; Chen *et al*, 2007). Conversely, DAMPS released from injured or necrotic keratinocytes may be recognised by TLRs on immune cells in the microenvironment leading to signals that disrupt the anti-tumour response leading to cancer progression (Lotze *et al*, 2007). The DAMP, HMGB1, has been found to be upregulated in some carcinomas in which it activates TLRs, including TLR2, on immune cells and enhances tumour progression (Ellerman *et al*, 2007).

This study found that 64% of malignant keratinocytes in OSCC and 74% of dysplastic keratinocytes in ED expressed TLR2, but that TLR2 was not expressed on the keratinocytes in the epithelium, which was hyperplastic in response to chronic irritation. To our knowledge, this is the first report demonstrating TLR2 expression on human oral cancer cells and dysplastic oral epithelial cells. Cancer cells from other sites (e.g., melanoma, breast) have been shown to express TLR2, and TLR4 expression has been noted on tumour cells in head and neck squamous cell carcinoma (Goto *et al*, 2008; Szczepanski *et al*, 2009; Xie *et al*, 2009). The expression of TLR2 on malignant keratinocytes suggests that they may be apoptotic resistant, as well as being able to induce apoptosis in targeted immune cells in the vicinity (Chen *et al*, 2007; Conroy *et al*, 2008; Huang *et al*, 2008). These cancer cells appear to have the ability to control the tumour microenvironment to their advantage and influence the immune cells present (Huang *et al*, 2005; He *et al*, 2007; Chen *et al*, 2008), hence, creating a pro-tumour outcome as proposed by Killeen *et al* (2006).

In conclusion, this study showed that TLR2 was expressed on the keratinocytes of dysplastic epithelium and OSCC. Positive TLR2 expression in the microenvironment suggests that immune surveillance is activated against the altered cells, whereas TLR2 expression by malignant keratinocytes may correlate with apoptosis resistance and, hence, the survival of tumour cells. Now that TLR2-positive cells have been detected in OSCC, this proposition needs to be tested to determine their functional activity in terms of gene expression and cytokine profiling.

## Figures and Tables

**Table 1:** Comparison of TLR2 expression in OSCC, ED and IH

**Lesion**	**TLR2 mean (%)**	**TLR2 mean rank**
OSCC	19.9	93.7
ED	16.4	80.8
IH	7.5	46.5

Abbreviations: ED=epithelial dysplasia; IH=irritative hyperplasia; OSCC=oral squamous cell carcinoma; TLR2=tumour like receptor 2.

OSCC *vs* IH, *P*<0.001 (Mann–Whitney's *U*-test).

ED *vs* IH, *P*<0.001 (Mann–Whitney's *U*-test).

OSCC *vs* ED, *P*>0.05 (Mann–Whitney's *U*-test).

**Table 2:** Expression of TLR2 by keratinocytes in OSCC, ED and IH

**Lesion**	**Negative (%)**	**Weakly positive (%)**	**Strongly positive (%)**
OSCC	36	26	38
ED	26	44	30
IH	100	—	—

Abbreviations: ED=epithelial dysplasia; IH=irritative hyperplasia; OSCC=oral squamous cell carcinoma.
